# Closed-Tube pH-Sensitive Loop-Mediated Isothermal Amplification for Rapid Detection of Tomato Leaf Curl New Delhi Virus in Vegetable Crops

**DOI:** 10.3390/plants15213352

**Published:** 2026-10-29

**Authors:** Xuling Zhai, Yixuan Lin, Wenrui Liu, Jinqiang Yan, Zhenqiang Cao, Jiesheng Hong, Piaoyun Sun, Qiancheng Mao, Biao Jiang

**Affiliations:** 1 Vegetable Research Institute, Guangdong Academy of Agricultural Sciences, Guangzhou 510640, China; zhaixuling@gdaas.cn (X.Z.);; 2 Guangdong Key Laboratory for New Technology Research of Vegetables, Guangzhou 510640, China

**Keywords:** tomato leaf curl New Delhi virus, pH-LAMP, rapid detection, field screening

## Abstract

Tomato leaf curl New Delhi virus (ToLCNDV) threatens cucurbit and solanaceous vegetable production, highlighting the need for rapid screening outside centralized laboratories. Here, we developed a closed-tube, pH-sensitive loop-mediated isothermal amplification (pH-LAMP) assay targeting a conserved region of ToLCNDV DNA-A. The assay incorporated six primers into a 25-µL reaction containing a weakly buffered master mix and a pH indicator. Incubation at 65 °C for 40 min produced a red-to-yellow color change that could be interpreted visually without opening the reaction tube. In a ten-fold dilution series of a ToLCNDV DNA-A plasmid, positive reactions were observed at concentrations ranging from 1000 ng µL^−1^ to 1 fg µL^−1^, with 1 fg µL^−1^ being the lowest tested concentration yielding a positive result. No cross-reactivity was observed with the five non-target vegetable viruses tested, and the healthy wax gourd control remained negative. In field screening, all 17 samples selected on the basis of symptoms from seven crop species tested positive by both pH-LAMP and conventional PCR, yielding 100% positive agreement in this selected symptomatic sample set. An additional 16 asymptomatic samples collected from plants of four crop species growing near symptomatic plants were screened, of which 11 yielded positive pH-LAMP results. These proof-of-concept results support the potential of closed-tube pH-LAMP for rapid, preliminary ToLCNDV screening using portable heating equipment and visual readout without post-amplification tube opening.

## 1. Introduction

Tomato leaf curl New Delhi virus (ToLCNDV) is a bipartite begomovirus with circular single-stranded DNA-A and DNA-B components. A foundational study established its bipartite genome organization and showed that coat protein was dispensable for experimental infectivity [1]. ToLCNDV is transmitted principally by *Bemisia tabaci* and infects a broad range of cultivated and wild hosts, with particularly damaging disease in cucurbits and solanaceous vegetables [2,3]. Its expansion beyond South Asia, including the Mediterranean emergence of the ToLCNDV-ES strain, illustrates the capacity of this virus complex to adapt, recombine and invade new production systems [4,5,6,7]. Biological comparisons further show host- and isolate-dependent differences in accumulation and pathogenicity [8,9,10], complicating symptom-based diagnosis. ToLCNDV infection can cause severe leaf yellowing and curling, stunting and impaired plant development in tomato and cucurbit crops; experimental infections have also caused necrosis and death in susceptible melon plants [9]. These effects on crop health, together with the potential for symptomless infection in some host–isolate combinations [8,9,10], underline the need for rapid detection to support surveillance and timely management.

Timely and specific detection is essential for surveillance, clean-plant programs, vector management and the removal of infection foci. However, leaf curling, mosaic, yellowing, stunting and fruit deformation are not pathognomonic because they can be caused by other viruses, mixed infections, arthropod injury or abiotic stress. Conventional PCR and quantitative PCR (qPCR) are sensitive and sequence-specific, and validated ToLCNDV qPCR and multiplex PCR protocols are available [11,12,13]. Routine qPCR requires thermocyclers with optical detection, while conventional PCR requires thermal cycling and a way to analyze amplicons. Antibody-based methods [14,15], optical-fiber localized surface plasmon resonance sensing [16], and CRISPR-associated detection [17,18] expand the diagnostic toolkit, but each introduces specific requirements for reagents, instrumentation or workflow control.

Loop-mediated isothermal amplification (LAMP) uses a strand-displacing polymerase and four to six primers to recognize multiple regions of a target sequence, enabling amplification at a single temperature [19,20,21]. LAMP assays have previously been developed for ToLCNDV detection in potato and watermelon [22,23]. In 2023, Caruso et al. [24] reported an in-field real-time LAMP assay that combined fluorescence monitoring with rapid sample preparation. Building on these developments, pH-sensitive endpoint detection offers an alternative approach to simplifying result interpretation. A pH indicator added before amplification enables direct visual detection of a color change without an optical reader or post-amplification tube opening [25]. This format therefore provides an opportunity to reduce endpoint-reading equipment requirements and post-amplification handling.

Here, we developed a closed-tube pH-sensitive LAMP assay targeting a conserved region of ToLCNDV DNA-A for rapid visual screening. As an assay-development proof of concept, we evaluated its performance using ToLCNDV-positive plant material, a plasmid dilution series, and five non-target vegetable viruses. We also compared portable-heater pH-LAMP with conventional PCR in 17 symptomatic field samples from seven crop species and explored its screening potential in 16 asymptomatic samples from four crop species collected near symptomatic plants. The study focused on the feasibility of closed-tube visual detection, with field results interpreted within the limits of these selected sample sets. Because the workflow retained CTAB-based DNA extraction, its practical simplification centered on amplification and readout; integration with rapid sample preparation and broader diagnostic validation remain necessary for routine field application.

## 2. Results

### 2.1. Comparative Genomic Analysis Guided the Selection of a Conserved DNA-A Target

**Figure 1:**
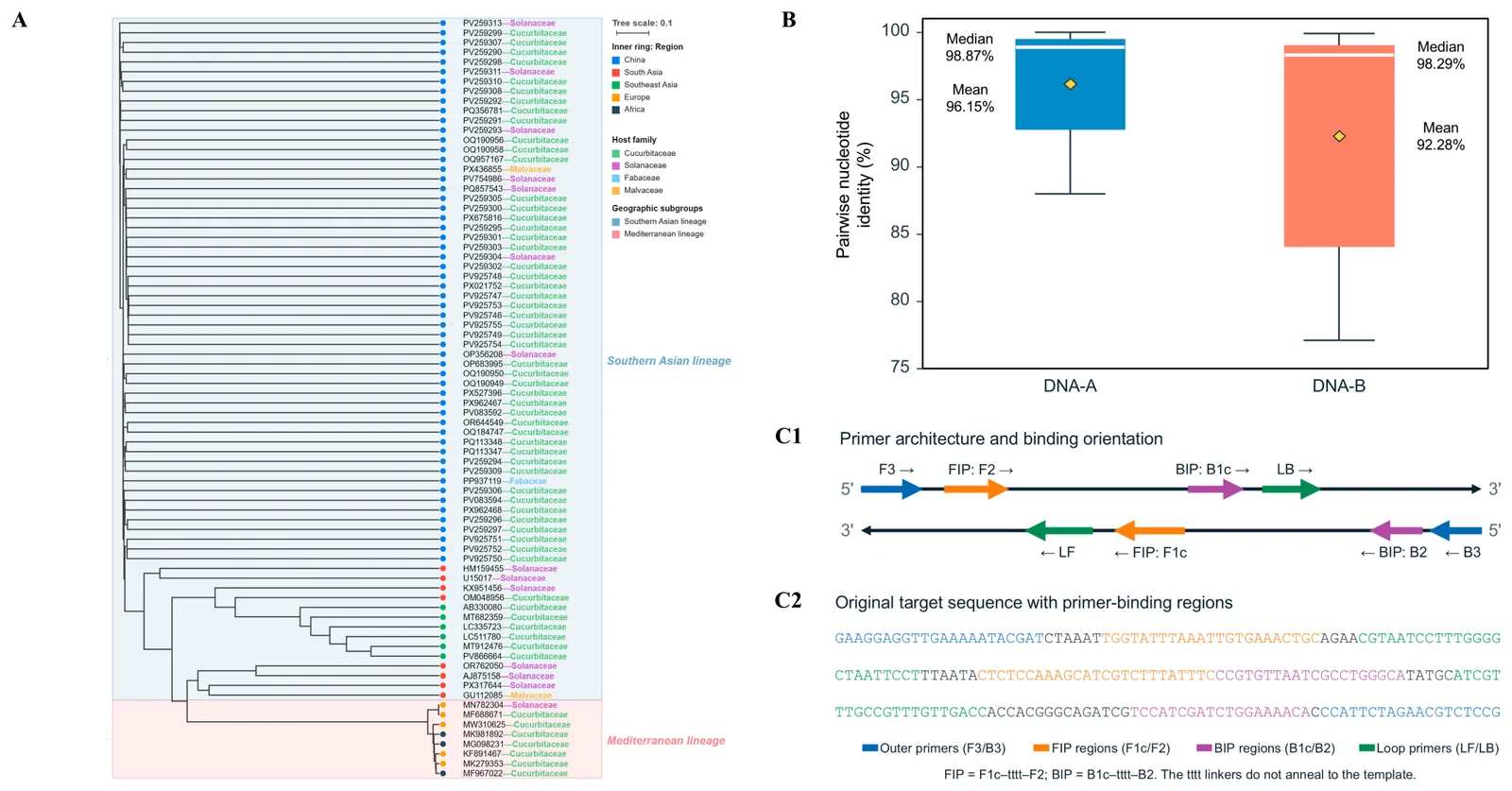
Comparative genomic analysis and selection of the ToLCNDV pH-LAMP target region. (**A**) Unrooted neighbor-joining tree based on complete DNA-B sequences of 78 ToLCNDV isolates from different geographic regions and host families. Colored tip symbols indicate geographic origin, colored accession labels indicate host family, and shaded backgrounds denote the Southern Asian and Mediterranean lineages. (**B**) Comparison of pairwise nucleotide identities between the complete DNA-A and DNA-B sequences of the 78 ToLCNDV isolates. Boxes represent the interquartile range (IQR; 25th–75th percentiles), the horizontal line within each box indicates the median, and yellow diamonds indicate the mean. The mean and median pairwise identity values are indicated for each genomic component. (**C1**) Binding positions and orientations of the six LAMP primers within a conserved 210-bp region (nt 2055–2264 of GenBank accession PQ356780) in the *AC1* coding sequence. F3 and B3 are the outer primers, FIP and BIP are the inner primers, and LF and LB are the loop primers. (**C2**) Nucleotide sequence of the target region showing the corresponding primer-binding sites. The four-thymidine (tttt) linkers incorporated into FIP and BIP serve as non-complementary spacer sequences and therefore do not anneal to the target sequence.

To improve the predicted inclusivity of the pH-LAMP assay, we analyzed 78 ToLCNDV isolate sequences representing diverse crop hosts and geographic regions worldwide. Phylogenetic analysis resolved these isolates into two major groups corresponding broadly to the Southern Asian and Mediterranean lineages (Figure 1A), consistent with the population structure reported previously [4,9,10]. Isolates from different crop families occurred within the same major lineages, indicating that phylogenetic clustering was associated more strongly with geographic origin than with host species.

Across the 78 isolates, the mean pairwise nucleotide identities were 96.15% for DNA-A and 92.28% for DNA-B, with corresponding medians of 98.87% and 98.29%, respectively (Figure 1B and Figure S1). Each component comprised 3003 unique pairwise comparisons (78 × 77/2). The mean identity of DNA-A was 3.87 percentage points higher than that of DNA-B, supporting the selection of DNA-A for primer design. Using the DNA-A sequence of the Guangdong wax gourd isolate PQ356780 as the reference [26], a conserved 210-bp region spanning nucleotides 2055–2264 within the *AC1* coding sequence was selected as the target. Six LAMP primers—F3, B3, FIP, BIP, LF, and LB—were designed to recognize eight distinct sites within this region (Figure 1(C1,C2)), with their arrangement and orientation conforming to the canonical LAMP primer architecture.

### 2.2. Closed-Tube pH-LAMP Produced a Clear, High-Contrast Visual Endpoint

**Figure 2:**
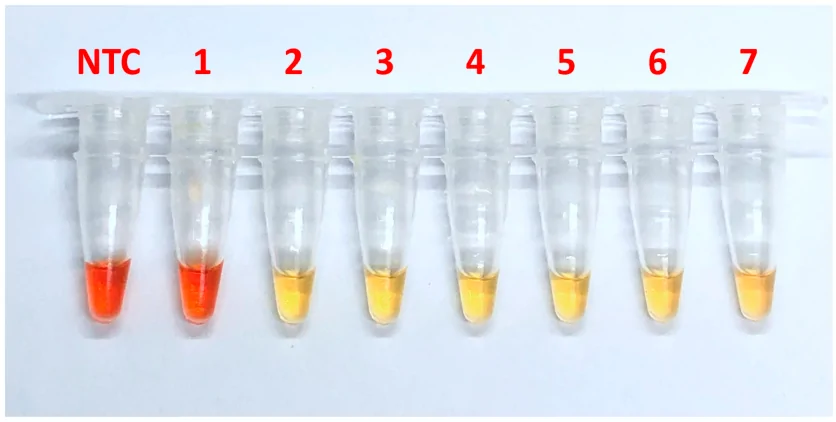
Visual endpoint of the closed-tube ToLCNDV pH-LAMP assay. NTC, no-template control; tube 1, DNA from a healthy plant; tubes 2–7, DNA from ToLCNDV-infected plants. Yellow indicates a positive result, whereas red indicates a negative result. The image shows a representative result from three independent experimental runs; the complete results are provided in Table S1.

Following incubation at 65 °C for 40 min, all six reactions containing DNA from ToLCNDV-infected plants changed from red to bright yellow, whereas the no-template control (NTC) and healthy-plant DNA control remained red (Figure 2). The results were assessed visually without opening the reaction tubes, thereby minimizing the risk of post-amplification contamination. Yellow and red were defined as the positive and negative endpoints, respectively, for subsequent analytical and field evaluations.

### 2.3. The Lowest Plasmid Concentration Yielding a Positive Result Was 1 fg µL^−1^

**Figure 3:**
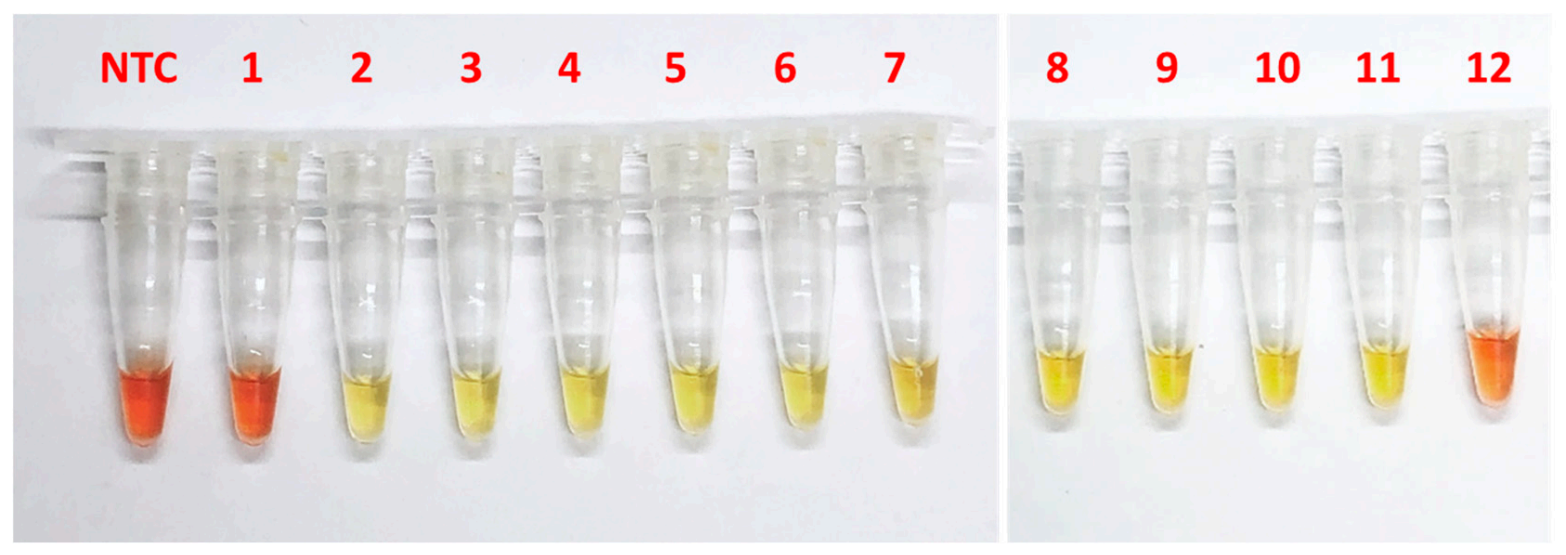
Detection of serially diluted ToLCNDV DNA-A plasmid by pH-LAMP. NTC, no-template control; tube 1, healthy plant DNA; tubes 2–12, DNA-A plasmid at 1000 ng, 100 ng, 10 ng, 1 ng, 100 pg, 10 pg, 1 pg, 100 fg, 10 fg, 1 fg and 0.1 fg µL^−1^, respectively. Tubes containing 1 fg µL^−1^ or higher concentrations changed from red to yellow, whereas the tube containing 0.1 fg µL^−1^ remained red. The image shows a representative result from three independent experimental runs; the complete results are provided in Table S2.

Across three independent experimental runs, the plasmid containing the ToLCNDV AC1 target fragment produced a red-to-yellow color change at each concentration from 1000 ng µL^−1^ to 1 fg µL^−1^, with all three reactions at each concentration testing positive (3/3, 100%). At 0.1 fg µL^−1^, all three reactions remained red, giving a positivity rate of 0% (0/3) (Figure 3; Table S2). The no-template control (NTC) and healthy-plant DNA control remained red in all three runs. Thus, under the conditions tested, 1 fg µL^−1^ was the lowest plasmid concentration yielding a positive result.

### 2.4. No Cross-Reactivity Was Observed with Five Non-Target Vegetable Viruses

**Figure 4:**
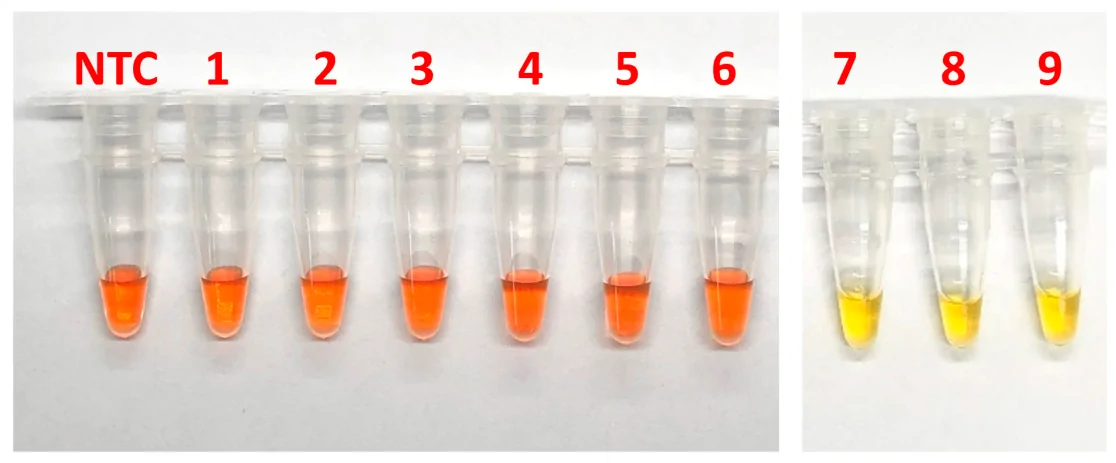
Evaluation of pH-LAMP cross-reactivity within the tested non-target virus panel. NTC, no-template control; tube 1, DNA from a healthy plant; tubes 2–6, nucleic acid templates derived from plants infected with SLCCNV, CMV, ZYMV, WMV, and CGMMV, respectively; tubes 7–9, DNA from ToLCNDV-infected plants. SLCCNV and ToLCNDV were tested using DNA, whereas the four RNA viruses (CMV, ZYMV, WMV, and CGMMV) were tested using cDNA. The image shows a representative result from three independent experimental runs; the complete results are provided in Table S3.

Only reactions containing the three ToLCNDV-positive templates changed from red to yellow. In contrast, reactions containing nucleic acids from plants infected with squash leaf curl China virus (SLCCNV), cucumber mosaic virus (CMV), zucchini yellow mosaic virus (ZYMV), watermelon mosaic virus (WMV), or cucumber green mottle mosaic virus (CGMMV) remained red, as did the no-template control (NTC) and healthy-plant DNA control (Figure 4). These results indicated that no cross-reactivity was detected with any of the five non-target viruses tested.

### 2.5. pH-LAMP Detected All Conventional PCR-Positive Symptomatic Field Samples and Identified ToLCNDV in Asymptomatic Plants

**Figure 5:**
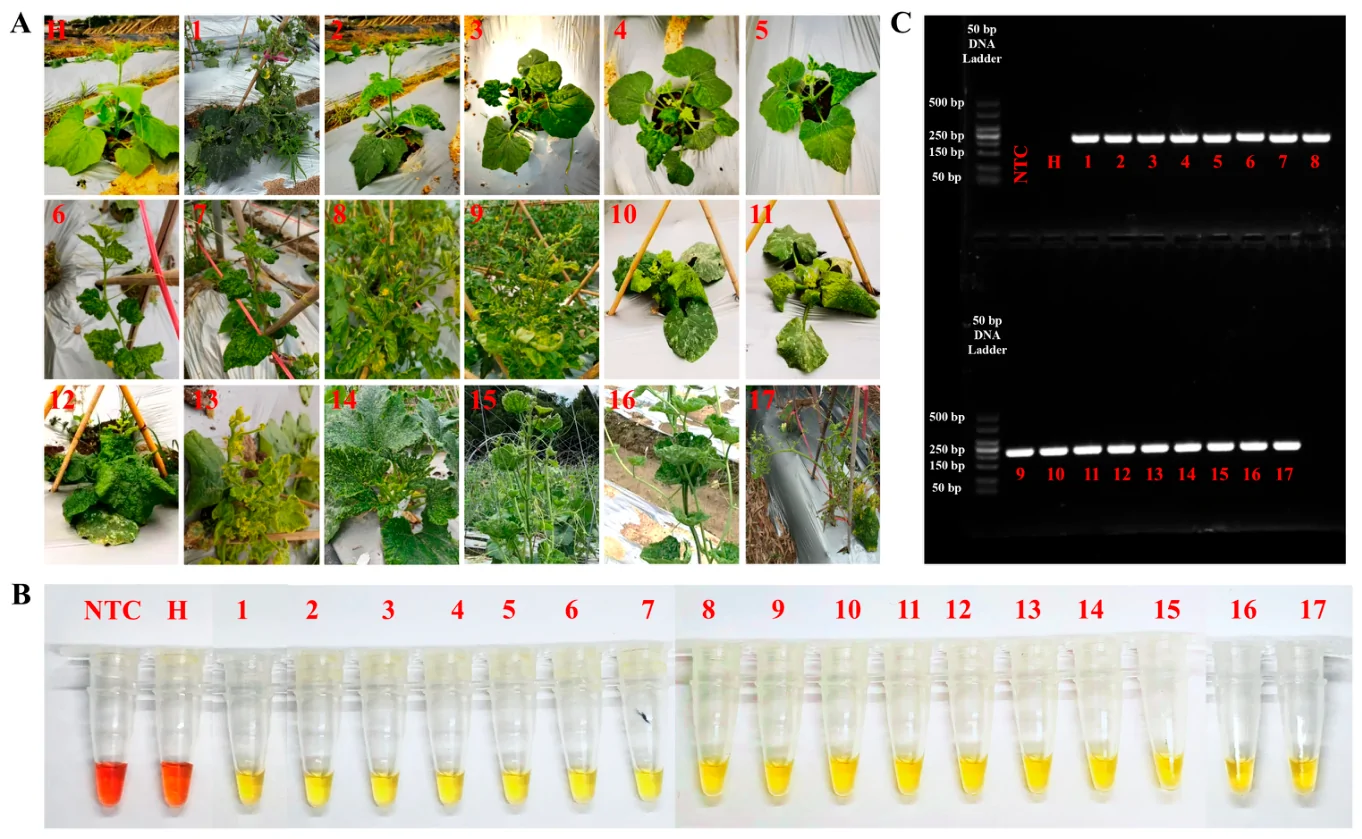
Comparison of conventional PCR and pH-LAMP results for symptomatic field samples. (**A**) Representative phenotypes of the healthy wax gourd control (H) and 17 symptomatic field samples: wax gourd (1–5), cucumber (6, 7), tomato (8, 9), pumpkin (10–13), zucchini (14), sponge gourd (15, 16), and bottle gourd (17). (**B**) Closed-tube pH-LAMP results. NTC and H remained red (negative), whereas samples 1–17 turned yellow (positive). The pH-LAMP assays were performed in three independent runs with consistent results; results from one run are shown. (**C**) Agarose gel electrophoresis of conventional PCR products. The upper gel shows the no-template control (NTC), H, and samples 1–8; the lower gel shows samples 9–17.

All 17 symptomatic field samples collected from seven crop species (wax gourd, cucumber, tomato, pumpkin, zucchini, sponge gourd, and bottle gourd) produced a red-to-yellow color change in the pH-LAMP assay using a portable heating block, whereas the healthy wax gourd DNA control and no-template control (NTC) remained red (Figure 5A,B). Conventional PCR also yielded positive results for all 17 symptomatic samples (Figure 5C). Both methods therefore identified 17 positive and zero negative samples, giving a positive agreement of 100% (17/17) within this selected symptomatic sample set.

An additional 16 asymptomatic samples from wax gourd, cucumber, tomato, and pumpkin plants growing near symptomatic plants were tested by both pH-LAMP and conventional PCR. Both methods identified 11 positive and five negative samples, yielding a positivity rate of 68.75% (11/16) and complete agreement in this group (Figure S3). Across the symptomatic and asymptomatic groups, both methods identified 28 positive and five negative samples among the 33 field samples, giving an overall agreement of 100% (33/33). The pH-LAMP results were consistent across three independent runs, and no intermediate colors or unscorable reactions were observed (Table S4).

## 3. Discussion

The practical contribution of this study is the development of a closed-tube visual screening assay that combines isothermal amplification with a pH-dependent colorimetric endpoint. Although previously reported ToLCNDV LAMP methods have provided alternatives to conventional thermal cycling [22,23,24], the present assay allows direct visual interpretation without an optical reader or post-amplification tube opening, simplifying readout and reducing handling. Keeping reaction tubes closed may also reduce opportunities for amplicon carryover contamination [27], although this benefit was not experimentally quantified in this study. These features support the potential use of the assay for preliminary screening in settings with limited laboratory equipment [28]. However, the current workflow relies on laboratory-based CTAB DNA extraction and therefore does not constitute a fully field-deployable sample-to-result system. Integration with a simplified sample preparation method, followed by validation under field conditions, would strengthen its suitability for routine use.

Comparative genomic analysis of 78 ToLCNDV isolates provided a rationale for selecting the amplification target. DNA-A had higher mean and median pairwise nucleotide identity than DNA-B, supporting selection of a conserved *AC1* region for primer design. However, overall genomic conservation does not necessarily indicate complete conservation of individual primer-binding sites or guarantee successful amplification of all geographically diverse isolates. Although the sequence analysis suggests potential compatibility with genetically diverse ToLCNDV isolates, experimental validation across different geographic lineages remains necessary, particularly considering the genetic diversity and evolutionary variation previously reported for this virus [29,30].

The pH-LAMP assay detected the plasmid containing the ToLCNDV *AC1* target fragment at concentrations as low as 1 fg µL^−1^ under the conditions tested. This represents the lowest concentration yielding a positive result in the tested dilution series rather than a statistically established limit of detection at a defined detection probability. Conventional PCR was employed to evaluate agreement with pH-LAMP using naturally infected field samples, but a parallel analytical-sensitivity comparison using identical plasmid dilution series was not performed. This distinction is important because viral loads in naturally infected samples can vary considerably depending on host species, infection stage, tissue type, and other biological factors. Consequently, serial dilution of different field samples without prior quantification and normalization would not provide a sufficiently standardized basis for directly comparing the analytical sensitivity of the two methods. The present study therefore focused on evaluating detection agreement in naturally infected samples rather than conducting a quantitative comparison of analytical sensitivity. A rigorous comparison would require both assays to be evaluated using the same quantified DNA template, standardized template inputs, and consistent positivity criteria. Accordingly, the present findings do not establish whether pH-LAMP has analytical sensitivity equivalent or superior to conventional PCR. Comparisons with previously reported detection limits should also consider differences in template type, input volume, concentration units, and criteria for defining positive reactions.

No cross-reactivity was observed with the five non-target viral template preparations tested. SLCCNV was the only non-target begomovirus in this panel; its inclusion was relevant because SLCCNV and ToLCNDV have both been documented in cucurbit crops in Guangdong Province [26,31]. The other four challenges were cDNA preparations from plants infected with RNA viruses. Negative reactions in this limited panel do not establish complete species-level exclusivity. Broader testing should include additional closely related begomoviruses, mixed infections and symptomatic plants confirmed negative for ToLCNDV, with independently verified template identity and adequate template input.

The field evaluation further demonstrated the potential utility of the assay for detecting ToLCNDV in both symptomatic and asymptomatic plants. All 17 symptomatic samples collected from seven crop species were positive by both pH-LAMP and conventional PCR, yielding 100% (17/17) positive agreement within this selected sample set. This agreement demonstrates consistent detection in the samples examined but should not be interpreted as an estimate of diagnostic sensitivity or overall accuracy in an unselected field population. Because no PCR-negative symptomatic samples were obtained, diagnostic specificity could not be determined from this subset. An additional 16 asymptomatic samples were collected from wax gourd, cucumber, tomato, and pumpkin plants growing near symptomatic plants. Both methods identified 11 positive and five negative samples, showing complete agreement in this group. However, PCR amplicons were not sequenced, and both assays targeted the same *AC1* region. Their agreement therefore remains susceptible to shared target-region bias and does not constitute confirmation at an independent genomic locus. These findings suggest that the assay may help identify potentially infected plants overlooked during symptom-based field inspections and support proactive surveillance in vegetable production systems. Nevertheless, the asymptomatic samples were collected near symptomatic plants rather than through random population sampling, so their positivity rate should not be interpreted as an estimate of infection prevalence among asymptomatic plants. Further evaluation using larger, more representative sample populations, including both PCR-positive and PCR-negative samples, is needed to establish diagnostic performance under diverse field conditions.

The reliability of the pH-dependent colorimetric endpoint also warrants consideration. pH-sensitive LAMP relies on amplification-associated changes in reaction pH under weakly buffered conditions [25], making visual interpretation potentially susceptible to template composition and reagent handling. Studies of other colorimetric LAMP systems have shown that buffered DNA eluates can reduce the color contrast between positive and negative reactions [32] and that acidic samples can induce color changes independently of amplification [33]. Atmospheric CO_2_ absorption has also been discussed as a potential source of reaction acidification [33]. These findings identify potential sources of interference, although the effects of buffer composition, plant extract acidity, and CO_2_ exposure were not experimentally evaluated in the present assay. As procedural precautions, DNA was eluted in nuclease-free water, template input volumes were standardized, and reactions were assembled on ice and kept closed throughout amplification and endpoint interpretation. No intermediate colors or unscorable reactions were observed, and results were consistent across three independent runs. Nevertheless, these observations do not establish robustness across different extract compositions or environmental conditions. Appropriate positive, healthy-plant, and no-template controls, together with a defined readout interval and criteria for handling ambiguous colors, remain important for reliable visual interpretation, particularly under field conditions.

Overall, this study establishes a proof of concept for a closed-tube, pH-sensitive LAMP assay for rapid visual screening of ToLCNDV. Under the conditions tested, the assay detected the target plasmid at concentrations as low as 1 fg µL^−1^, showed no detectable cross-reactivity with the five non-target viruses examined, and produced results fully concordant with conventional PCR across 33 field samples. These findings support the potential application of the assay in surveillance beyond symptom-based inspection. Nevertheless, the evaluation remains preliminary. Analytical sensitivity relative to conventional PCR, specificity against additional closely related begomoviruses, and diagnostic performance in larger, representative field populations require further investigation. Although results were consistent across three independent runs, broader assessments of robustness, within-run repeatability, and reproducibility across operators and laboratories are still needed. The current workflow also relies on laboratory-based CTAB DNA extraction. Integration with simplified sample preparation and further field validation would strengthen its practicality for routine ToLCNDV screening in agricultural settings with limited laboratory infrastructure.

## 4. Materials and Methods

### 4.1. Comparative Sequence Analysis and Primer Design

Comparative analysis of 78 ToLCNDV isolate sequences was used to compare DNA-A and DNA-B conservation and guide target selection. The DNA-A sequence of the Guangdong wax gourd isolate PQ356780 served as the reference [26]. The selected 210-bp region spans nucleotides 2055–2264, within the *AC1* coding sequence annotated on the complementary strand at nucleotides 1499–2584. Six LAMP primers were designed to recognize the canonical outer (F3/B3), inner (FIP/BIP) and loop (LF/LB) sites (Table 1). Oligonucleotides were synthesized by Sangon Biotech (Shanghai, China), diluted individually to 20 µM with nuclease-free water, and stored at −20 °C. GenBank DNA-A and DNA-B accession numbers, sequence lengths, isolate designations, geographic origins, hosts and recorded years for the 78 isolates are listed in Table S5.

### 4.2. Phylogenetic Analysis

Complete DNA-B nucleotide sequences from 78 ToLCNDV isolates representing different geographic regions and host families were aligned using ClustalW implemented in MEGA version 7.0 with default parameters [34]. The phylogenetic tree was reconstructed using the neighbor-joining (NJ) method. Coding and non-coding regions were included, and alignment gaps and missing data were treated using pairwise deletion. Branch support was assessed by nonparametric bootstrap analysis with 1000 replicates. The tree was retained as unrooted, without an outgroup or midpoint rooting.

### 4.3. Nucleic Acid Preparation

Total DNA was extracted from plant samples using the cetyltrimethylammonium bromide (CTAB) method described by Doyle and Doyle [35]. To minimize interference from excessive buffering with the pH-dependent colorimetric endpoint, DNA was eluted in nuclease-free water rather than a strongly buffered Tris-based solution. For the RNA viruses included in the analytical specificity panel, cDNA was synthesized using the TransScript^®^ One-Step gDNA Removal and cDNA Synthesis SuperMix Kit (TransGen Biotech, Beijing, China; Cat. No. AT311) according to the manufacturer’s instructions.

### 4.4. pH-Sensitive LAMP and Visual Interpretation

LAMP reactions were prepared using the Colorimetric pH-Sensitive LAMP Kit (TransGen Biotech, Beijing, China; Cat. No. LP321). Because Bst II DNA polymerase retains activity at room temperature, all reactions were assembled on ice. Each reaction had a total volume of 25 µL; reagent stock concentrations, component volumes and final concentrations are listed in Table 2. A no-template control (NTC) and a healthy-plant DNA control were included in each experiment.

The reaction tubes were incubated at 65 °C for 40 min using a metal heating block or water bath and subsequently cooled to room temperature. The tubes remained closed during result interpretation to minimize the risk of amplicon contamination. Color changes were assessed visually against a white background: yellow was scored as positive, whereas red was scored as negative or below the assay detection limit.

### 4.5. Analytical Sensitivity

A circular pGreenII 62-SK plasmid containing a 210-bp fragment of the ToLCNDV *AC1* coding sequence (GenBank accession PQ356780; Figure S2) was used to evaluate analytical sensitivity. The total plasmid length, including the insert, was 3542 bp. Plasmid DNA was quantified using a NanoDrop 2000 spectrophotometer (Thermo Fisher Scientific, Wilmington, DE, USA) and serially diluted ten-fold in nuclease-free water to obtain template concentrations of 1000 ng µL^−1^, 100 ng µL^−1^, 10 ng µL^−1^, 1 ng µL^−1^, 100 pg µL^−1^, 10 pg µL^−1^, 1 pg µL^−1^, 100 fg µL^−1^, 10 fg µL^−1^, 1 fg µL^−1^, and 0.1 fg µL^−1^. Each reaction contained 2 µL of template, and each dilution was tested in three independent experimental runs at 65 °C for 40 min. The estimated number of target copies per reaction was calculated as follows:

$$\mathrm{Target copies per reaction}=\frac{C\times V\times 6.022\times 10^{23}}{3542\times 660}$$

where *C* is the plasmid DNA concentration in g µL^−1^, *V* is the template volume per reaction (2 µL), and 660 g mol^−1^ bp^−1^ is the average molecular weight of double-stranded DNA per base pair. Each plasmid contained one copy of the target sequence. The dilution series therefore corresponded to approximately 5.15 × 10^11^ to 5.15 × 10^1^ target copies per reaction. A no-template control (NTC) and a healthy-plant DNA control were included in each experiment.

### 4.6. Evaluation of Cross-Reactivity

Cross-reactivity was evaluated using nucleic acids from plants infected with SLCCNV (genus *Begomovirus*), CMV (genus *Cucumovirus*), ZYMV (genus *Potyvirus*), WMV (genus *Potyvirus*), and CGMMV (genus *Tobamovirus*), together with three ToLCNDV-positive plant samples, a healthy-plant control, and a no-template control (NTC). Templates for ToLCNDV and the five non-target viruses were obtained from field-grown plants initially selected based on visible symptoms. Infection with the corresponding virus was subsequently confirmed by conventional PCR for the DNA viruses and RT-PCR for the RNA viruses.

### 4.7. Field Sample Testing and Comparison with Conventional PCR

The field evaluation included 33 young-leaf samples collected from Baiyun and Zengcheng districts, Guangzhou, China, between 18 May and 25 June 2025. Seventeen samples were selected based on visible symptoms suggestive of ToLCNDV infection. The remaining 16 were collected from plants growing near symptomatic plants and showed no visible symptoms of viral disease at the time of sampling (Table S4). Samples were stored and transported in liquid nitrogen, and total DNA was extracted in the laboratory using the CTAB method described in Section 4.3. DNA extracted from a healthy wax gourd plant served as the healthy-plant negative control. All 33 field samples and the healthy wax gourd control were tested by pH-LAMP at 65 °C for 40 min using a portable heating block powered by a power bank. Aliquots of the same DNA extracts were also tested for ToLCNDV by conventional PCR under standard laboratory conditions.

Conventional PCR targeted the same 210-bp region within the *AC1* coding sequence as the pH-LAMP assay, using the primers listed in Table S6. Each 20-µL PCR reaction contained 1 µL of DNA template, 10 µL of Taq PCR mix, 0.5 µL each of the forward and reverse primers, and 8 µL of nuclease-free water. The thermal cycling conditions were as follows: initial denaturation at 95 °C for 3 min; 35 cycles of denaturation at 94 °C for 25 s, annealing at 58 °C for 15 s, and extension at 72 °C for 20 s; and a final extension at 72 °C for 5 min, followed by a hold at 4 °C.

### 4.8. Data Analysis

All LAMP reactions were interpreted visually by personnel blinded to both the ToLCNDV infection status of the samples and the corresponding PCR results.

## 5. Conclusions

This study developed a closed-tube pH-LAMP assay for preliminary visual screening of ToLCNDV. Targeting a conserved DNA-A region, the assay enables visual interpretation after incubation at 65 °C for 40 min without post-amplification tube opening or specialized optical equipment. The lowest tested plasmid concentration yielding a positive result was 1 fg µL^−1^, equivalent to 2 fg per reaction, and no cross-reactivity was observed with the five non-target viruses tested. Results were consistent across three independent pH-LAMP runs. The assay showed complete agreement with conventional PCR across 33 field samples: all 17 symptomatic samples were positive, while the 16 asymptomatic samples included 11 positive and five negative samples by both methods. These findings support its potential for screening plants with or without visible symptoms. Further validation using independent genomic targets, additional closely related begomoviruses, and larger, representative field populations is needed. Broader assessments of robustness and reproducibility, together with integration of validated rapid sample preparation, would strengthen the assay’s suitability for routine field screening.

## Figures and Tables

**Table 1:** Primer sequences used for pH-sensitive LAMP detection of ToLCNDV.

Primer	Function	Sequence (5′–3′)	Coordinates on PQ356780
F3	Outer forward	GAAGGAGGTTGAAAAATACGAT	2055–2076 (+)
B3	Outer reverse	CGGAGACGTTCTAGAATGG	2246–2264 (−)
FIP	Inner forward	GGAAATAAAGACGATGCTTTGAGAGttttTGGTATTTAAATTGTGAAACTGC	F1c: 2140–2164 (−) F2: 2083–2105 (+)
BIP	Inner reverse	CGTGTTAATCGCCTGGGCATttttGTGTTTTCCAGATCGATGG	B1c: 2165–2184 (+) B2: 2226–2244 (−)
LF	Loop forward	AGGAATTAGCCCCAAAGGATTACG	2110–2133 (−)
LB	Loop reverse	TCGTTTGCCGTTTGTTGACCA	2190–2210 (+)

Lowercase tttt indicates the four-thymidine linker within FIP and BIP. Coordinates are 1-based and inclusive on GenBank reference PQ356780. (+) denotes identity to the displayed reference strand, and (−) denotes identity to its reverse complement. Coordinates for FIP and BIP are listed separately for their target-binding elements; the linkers have no genomic coordinates.

**Table 2:** Composition of the 25-µL pH-sensitive LAMP reaction.

Component	Stock	Volume (µL)	Final
2.5× pH-sensitive LAMP mix	2.5×	10.0	1×
N-Red pH indicator	—	1.7	—
F3 primer	20 µM	0.5	0.4 µM
B3 primer	20 µM	0.5	0.4 µM
FIP primer	20 µM	2.0	1.6 µM
BIP primer	20 µM	2.0	1.6 µM
LF primer	20 µM	1.0	0.8 µM
LB primer	20 µM	1.0	0.8 µM
Bst II DNA polymerase	8 U µL^−1^	1.0	8 U
Template DNA	—	2.0	—
Nuclease-free water	—	3.3	—
Total		25.0	

## Data Availability

The reported assay outcomes are presented in the article. Accession numbers and metadata for the 78 ToLCNDV isolates are provided in Supplementary Table S5; the corresponding sequence records are available through GenBank.

## Supplementary Materials

The following supporting information can be downloaded at: https://www.mdpi.com/article/10.3390/plants15213352/s1. Figure S1. Pairwise nucleotide-identity matrix of the complete DNA-A and DNA-B sequences from the 78 isolates. Figure S2. Map of the plasmid containing the ToLCNDV DNA-A sequence. Figure S3. Conventional PCR detection of ToLCNDV in 16 asymptomatic field samples. Table S1. Colorimetric results of the closed-tube ToLCNDV pH-LAMP assay across three independent experimental runs. Table S2. Template concentrations, estimated target copies per reaction, and replicate-level colorimetric results for the pH-LAMP analytical sensitivity assay and controls. Table S3. Colorimetric results of the pH-LAMP cross-reactivity evaluation across three independent experimental runs. Table S4. Comparison of pH-LAMP and conventional PCR results for 17 symptomatic and 16 asymptomatic field samples. Table S5. Details of the 78 ToLCNDV isolates included in the comparative genomic analysis. Table S6. Primer sequences used for conventional PCR detection of ToLCNDV.
