# Supplementary material for: Closed-Tube pH-Sensitive Loop-Mediated Isothermal Amplification for Rapid Detection of Tomato Leaf Curl New Delhi Virus in Vegetable Crops
Source: Plants (Basel). 2026 Oct 29;15(21):3352. doi: 10.3390/plants15213352 (PMC13765878; doi:10.3390/plants15213352)
Supplement: Supplementary file 1 [file plants-15-03352-s001.zip › plants-4609974-supplementary.pdf]

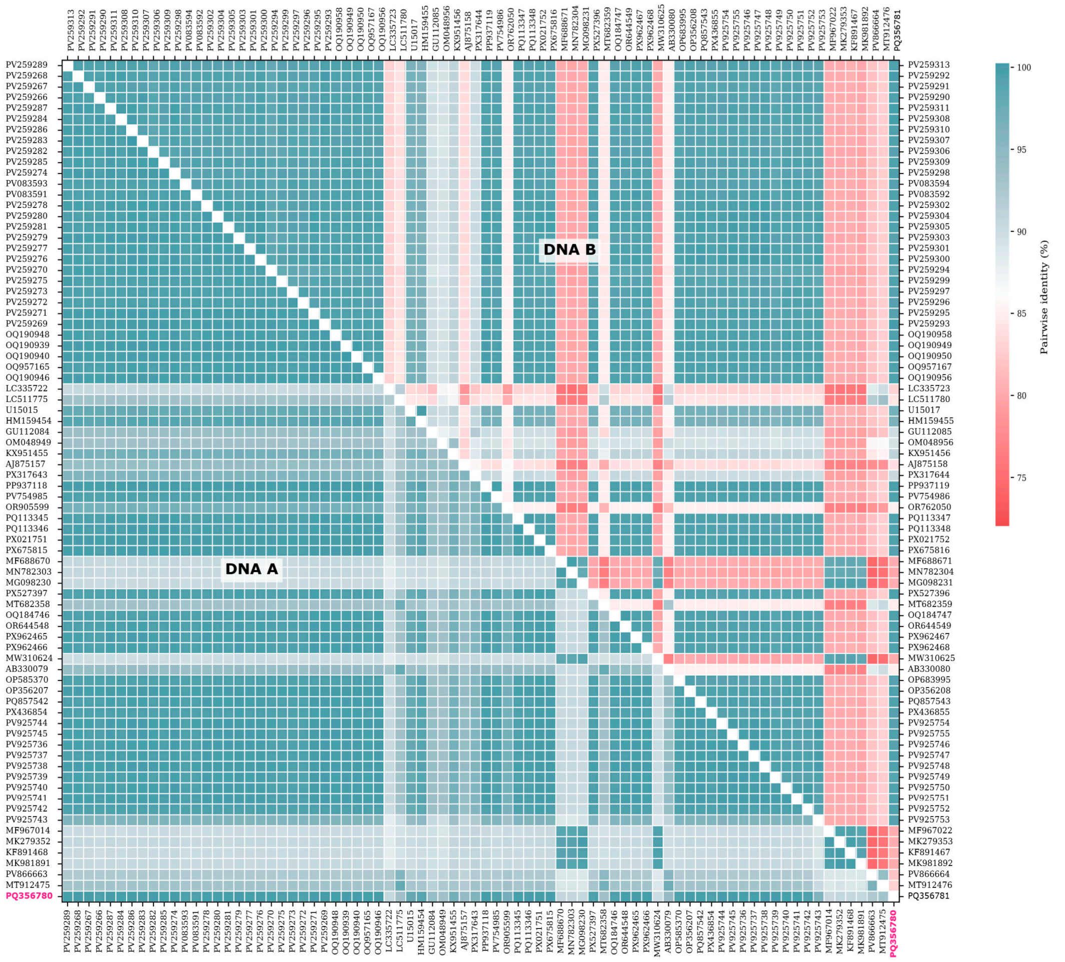

Figure S1. Pairwise nucleotide-identity matrix of the complete DNA-A and DNA-B sequences from the 78 isolates. The lower-left and upper-right triangles represent DNA-A and DNA-B, respectively, with nucleotide identity indicated by the color scale. The Guangdong wax gourd isolate used as the reference for primer design is highlighted in red.

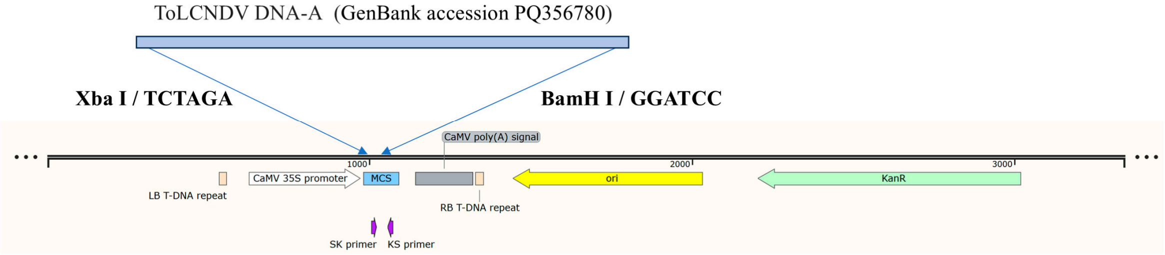

Figure S2. Map of the plasmid containing the ToLCNDV DNA-A sequence.

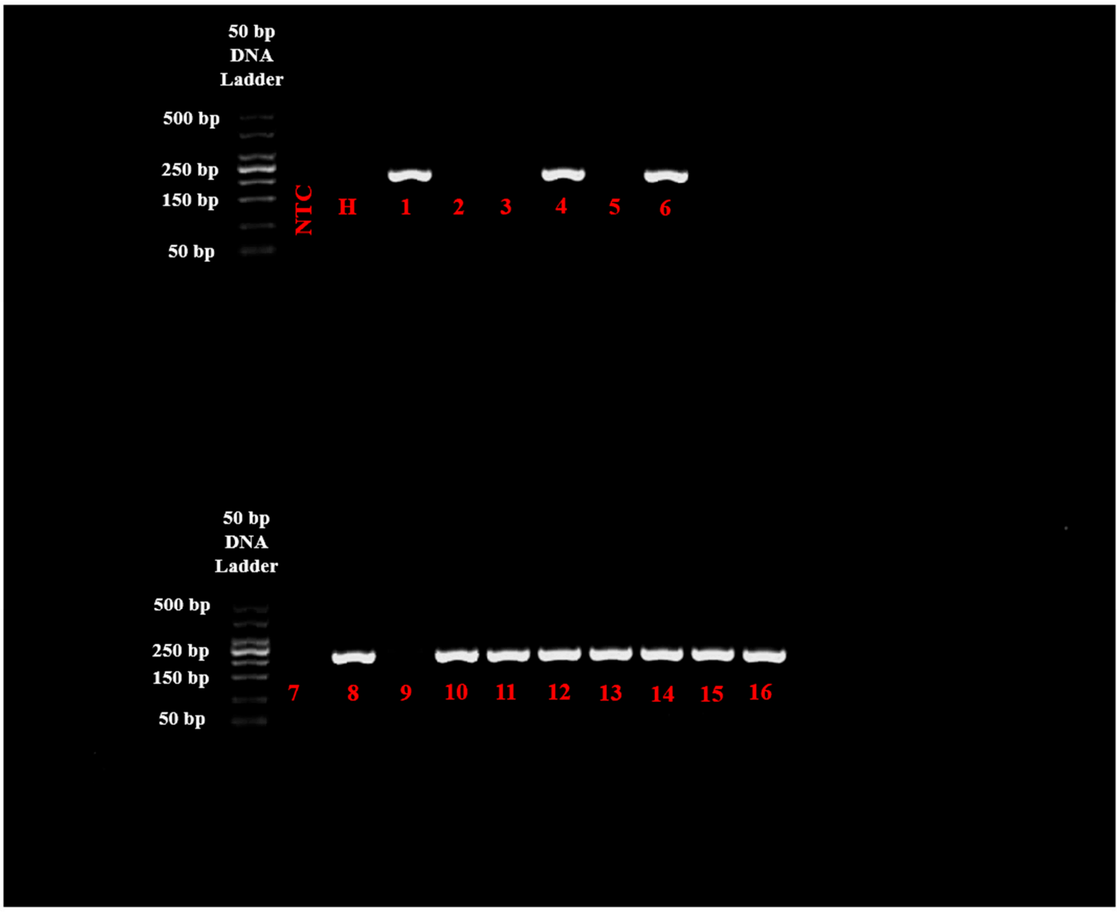

Figure S3. Conventional PCR detection of ToLCNDV in 16 asymptomatic field samples. Agarose gel electrophoresis of PCR products targeting a 210-bp region within the ToLCNDV *AC1* coding sequence. The upper gel shows the no-template control (NTC), healthy wax gourd control (H), and samples 1–6; the lower gel shows samples 7–16. A 50-bp DNA ladder was used as the molecular size marker. Samples 1, 4, 6, 8, and 10–16 yielded the expected amplicon, consistent with the pH-LAMP results reported in Supplementary Table S4.

Table S1. Colorimetric results of the closed-tube ToLCNDV pH-LAMP assay across three independent experimental runs.

Sample ID	Host species	Reaction color (runs 1/2/3)	pH-LAMP result
NTC	_	Red/Red/Red	Negative
1	Wax gourd	Red/Red/Red	Negative
2	Wax gourd	Yellow/Yellow/Yellow	Positive
3	Wax gourd	Yellow/Yellow/Yellow	Positive
4	Wax gourd	Yellow/Yellow/Yellow	Positive
5	Wax gourd	Yellow/Yellow/Yellow	Positive
6	Wax gourd	Yellow/Yellow/Yellow	Positive
7	Wax gourd	Yellow/Yellow/Yellow	Positive

Notes: NTC, no-template control; tube 1, DNA from a healthy plant; tubes 2–7, DNA from ToLCNDV-infected plants. Yellow indicates a positive result, whereas red indicates a negative result.

Table S2. Template concentrations, estimated target copies per reaction, and replicate-level colorimetric results for the pH-LAMP analytical sensitivity assay and controls.

Template concentration	DNA mass per reaction	Estimated target copies per reaction	Reaction color (runs 1/2/3)	Positive / total reactions
NTC/0 ng μL^{-1}	0 ng	_	Red/Red/Red	0/3
1/50 ng μL^{-1}	100 ng	_	Red/Red/Red	0/3
2/1,000 ng μL^{-1}	2,000 ng	5.15×10^{11}	Yellow/Yellow/Yellow	3/3
3/100 ng μL^{-1}	200 ng	5.15×10^{10}	Yellow/Yellow/Yellow	3/3
4/10 ng μL^{-1}	20 ng	5.15×10^{9}	Yellow/Yellow/Yellow	3/3
5/1 ng μL^{-1}	2 ng	5.15×10^{8}	Yellow/Yellow/Yellow	3/3
6/100 pg μL^{-1}	200 pg	5.15×10^{7}	Yellow/Yellow/Yellow	3/3
7/10 pg μL^{-1}	20 pg	5.15×10^{6}	Yellow/Yellow/Yellow	3/3
8/1 pg μL^{-1}	2 pg	5.15×10^{5}	Yellow/Yellow/Yellow	3/3
9/100 fg μL^{-1}	200 fg	5.15×10^{4}	Yellow/Yellow/Yellow	3/3
10/10 fg μL^{-1}	20 fg	5.15×10^{3}	Yellow/Yellow/Yellow	3/3
11/1 fg μL^{-1}	2 fg	5.15×10^{2}	Yellow/Yellow/Yellow	3/3
12/0.1 fg μL^{-1}	0.2 fg	5.15×10^{1}	Red/Red/Red	0/3

Notes: The circular pGreenII 62-SK plasmid was quantified using a NanoDrop spectrophotometer. Its total length was 3,542 bp, including one 210-bp ToLCNDV *AC1* insert. Each reaction contained 2 μL of template. Each dilution was tested in three independent experimental runs at 65 °C for 40 min. Estimated target copies per reaction = [DNA mass per reaction (g) × 6.022×10^{23}] / [3,542 × 660], assuming one target copy per plasmid.

Table S3. Colorimetric results of the pH-LAMP cross-reactivity evaluation across three independent experimental runs.

Sample ID	Virus species	Host species	Reaction color (runs 1/2/3)	pH-LAMP result
NTC	_	_	Red/Red/Red	Negative
1	_	Wax gourd	Red/Red/Red	Negative
2	SLCCNV	Pumpkin	Yellow/Yellow/Yellow	Positive
3	CMV	Cucumber	Yellow/Yellow/Yellow	Positive
4	ZYMV	Zucchini	Yellow/Yellow/Yellow	Positive
5	WMV	Cucumber	Yellow/Yellow/Yellow	Positive
6	CGMMV	Cucumber	Yellow/Yellow/Yellow	Positive
7	ToLCNDV	Wax gourd	Yellow/Yellow/Yellow	Positive
8	ToLCNDV	Wax gourd	Yellow/Yellow/Yellow	Positive
9	ToLCNDV	Wax gourd	Yellow/Yellow/Yellow	Positive

Notes: NTC, no-template control; tube 1, DNA from a healthy plant; tubes 2–6, nucleic acid templates derived from plants infected with SLCCNV, CMV, ZYMV, WMV, and CGMMV, respectively; tubes 7–9, DNA from ToLCNDV-infected plants.

Table S4. Comparison of pH-LAMP and conventional PCR results for 17 symptomatic and 16 asymptomatic field samples.

Sample ID	Host species	Reaction color (runs 1/2/3)	pH-LAMP result	PCR result	Group
NTC	_	Red/Red/Red	Negative	Negative	
H	Wax gourd	Red/Red/Red	Negative	Negative	
1	Wax gourd	Yellow/Yellow/Yellow	Positive	Positive	Symptomatic group
2	Wax gourd	Yellow/Yellow/Yellow	Positive	Positive	
3	Wax gourd	Yellow/Yellow/Yellow	Positive	Positive	
4	Wax gourd	Yellow/Yellow/Yellow	Positive	Positive	
5	Wax gourd	Yellow/Yellow/Yellow	Positive	Positive	
6	Cucumber	Yellow/Yellow/Yellow	Positive	Positive	
7	Cucumber	Yellow/Yellow/Yellow	Positive	Positive	
8	Tomato	Yellow/Yellow/Yellow	Positive	Positive	
9	Tomato	Yellow/Yellow/Yellow	Positive	Positive	
10	Pumpkin	Yellow/Yellow/Yellow	Positive	Positive	
11	Pumpkin	Yellow/Yellow/Yellow	Positive	Positive	
12	Pumpkin	Yellow/Yellow/Yellow	Positive	Positive	
13	Pumpkin	Yellow/Yellow/Yellow	Positive	Positive	
14	Zucchini	Yellow/Yellow/Yellow	Positive	Positive	
15	Sponge gourd	Yellow/Yellow/Yellow	Positive	Positive	
16	Sponge gourd	Yellow/Yellow/Yellow	Positive	Positive	
17	Bottle gourd	Yellow/Yellow/Yellow	Positive	Positive	
NTC	_	Red/Red/Red	Negative	Negative	
H	Wax gourd	Red/Red/Red	Negative	Negative	

1	Wax gourd	Yellow/Yellow/Yellow	Positive	Positive	Asymptomatic group
2	Wax gourd	Red/Red/Red	Negative	Negative	
3	Wax gourd	Red/Red/Red	Negative	Negative	
4	Wax gourd	Yellow/Yellow/Yellow	Positive	Positive	
5	Cucumber	Red/Red/Red	Negative	Negative	
6	Cucumber	Yellow/Yellow/Yellow	Positive	Positive	
7	Cucumber	Red/Red/Red	Negative	Negative	
8	Cucumber	Yellow/Yellow/Yellow	Positive	Positive	
9	Tomato	Red/Red/Red	Negative	Negative	
10	Tomato	Yellow/Yellow/Yellow	Positive	Positive	
11	Tomato	Yellow/Yellow/Yellow	Positive	Positive	
12	Tomato	Yellow/Yellow/Yellow	Positive	Positive	
13	Pumpkin	Yellow/Yellow/Yellow	Positive	Positive	
14	Pumpkin	Yellow/Yellow/Yellow	Positive	Positive	
15	Pumpkin	Yellow/Yellow/Yellow	Positive	Positive	
16	Pumpkin	Yellow/Yellow/Yellow	Positive	Positive	

Table S5. Details of the 78 ToLCNDV isolates included in the comparative genomic analysis.

Continent	Geographic origin	Isolate	Year	Host	GenBank accession (DNA-A)	DNA-A length (nt)	GenBank accession (DNA-B)	DNA-B length (nt)
Asia	Anhui, China	HF23CB	2024	Common bean	PP937118	2739	PP937119	2693
Asia	Shanghai, China	8066	2023	Cucumber	OQ190946	2739	OQ190956	2693
Asia	Zhejiang, China	Zhejiang	2022	Tomato	OP356207	2739	OP356208	2693
Asia	Shanghai, China	0823-2	2023	Melon	OQ190940	2739	OQ190950	2693
Asia	Shanghai, China	0823-1	2023	Melon	OQ190939	2739	OQ190949	2693
Asia	Shanghai, China	7922	2023	Luffa	OQ190948	2739	OQ190958	2693
Asia	Shanghai, China	SH-WM1	2023	Watermelon	OQ957165	2739	OQ957167	2693
Asia	Jiangsu, China	22LHW	2023	Watermelon	OR644548	2739	OR644549	2691
Asia	Jiangsu, China	Haimen6	2023	Melon	OQ184746	2739	OQ184747	2692
Asia	Zhejiang, China	Ningbo6	2022	Melon	OP585370	2739	OP683995	2693
Asia	India	New Delhi	2010	Tomato	HM159454	2739	HM159455	2696
Asia	India	Not reported	2003	Tomato	U15015	2739	U15017	2696
Asia	India	KAN-6	2016	Potato	KX951455	2739	KX951456	2693
Asia	Pakistan	Khanewal23	2023	Tomato	OR905599	2739	OR762050	2692
Asia	India	OY135	2012	Okra	GU112084	2743	GU112085	2692
Asia	Thailand	cucumber: Thailand	2009	Cucumber	AB330079	2739	AB330080	2694
Asia	Bangladesh	Jessore	2006	Tomato	AJ875157	2740	AJ875158	2688
Asia	India	BG3-JnP	2022	Bitter gourd	OM048949	2740	OM048956	2696
Asia	Indonesia	BACu-20	2021	Cucumber	LC511775	2739	LC511780	2680
Asia	Cambodia	Cs2	2023	Cucumber	MT682358	2739	MT682359	2685
Asia	Malaysia	16MY1	2022	Melon	MT912475	2739	MT912476	2690
Asia	Indonesia	Indonesia	2018	Melon	LC335722	2740	LC335723	2684
Europe	Italy	Cum-45/16	2017	Pumpkin	MF688670	2738	MF688671	2686
Europe	Italy	Som-166/16	2020	Eggplant	MN782303	2738	MN782304	2686
Europe	Spain	ES13-35	2019	Zucchini	MK279352	2738	MK279353	2684
Africa	Algeria	Biskra	2020	Cucumber	MK981891	2738	MK981892	2684
Africa	Morocco	Agadir	2018	Pumpkin	MG098230	2738	MG098231	2684
Europe	Spain	Spain-Almeria	2015	Zucchini	KF891468	2738	KF891467	2684
Africa	Tunisia	D5	2019	Cucumber	MF967014	2738	MF967022	2684
Europe	France	CD20001	2021	Bottle gourd	MW310624	2738	MW310625	2683
Asia	Zhejiang, China	ZJ	2026	Pepper	PQ857542	2739	PQ857543	2693
Asia	Shenyang, China	JS	2026	Melon	PX962466	2739	PX962468	2692
Asia	Shenyang, China	HB	2026	Melon	PX962465	2739	PX962467	2693
Asia	Guangxi, China	GXGL-LHG1	2026	Monk fruit	PX675815	2739	PX675816	2693
Asia	Guangxi, China	GXBH-TG	2026	Melon	PX021751	2739	PX021752	2692
Asia	Malaysia	JC01	2025	Melon	PV866663	2739	PV866664	2692
Asia	Hubei, China	JZ	2025	Velvetleaf	PX436854	2739	PX436855	2692
Asia	Xinjiang, China	Turpan	2026	Melon	PX527397	2739	PX527396	2693
Asia	Bangladesh	po-Bd	2025	Potato	PX317643	2740	PX317644	2693
Asia	Guangdong, China	WLSG01	2025	Luffa	PQ113346	2739	PQ113348	2693
Asia	Guangdong, China	YLSG01	2025	Luffa	PQ113345	2739	PQ113347	2693
Asia	Guangxi, China	GXBHm106	2025	Melon	PV925745	2739	PV925755	2694
Asia	Guangxi, China	GXBHm105	2025	Melon	PV925744	2739	PV925754	2695
Asia	Guangxi, China	GXBHm76	2025	Melon	PV925743	2739	PV925753	2694
Asia	Guangxi, China	GXBHm34	2025	Melon	PV925742	2739	PV925752	2693
Asia	Guangxi, China	GXBHm32	2025	Melon	PV925741	2739	PV925751	2692
Asia	Guangxi, China	GXBHm19	2025	Melon	PV925740	2739	PV925750	2694
Asia	Guangxi, China	GXBHm18	2025	Melon	PV925739	2739	PV925749	2694
Asia	Guangxi, China	GXBHm14	2025	Melon	PV925738	2739	PV925748	2693
Asia	Guangxi, China	GXBHm13	2025	Melon	PV925737	2739	PV925747	2693
Asia	Guangxi, China	GXBHm10	2025	Melon	PV925736	2739	PV925746	2693
Asia	Anhui, China	FN	2025	Pepper	PV754985	2739	PV754986	2693
Asia	Guangdong, China	23GD_BeHi1	2025	Wax gourd	PQ356780	2739	PQ356781	2693
Asia	Guangdong, China	BYFQ	2025	Tomato	PV259289	2739	PV259313	2692
Asia	Guangdong, China	FSFQ	2025	Tomato	PV259287	2738	PV259311	2693
Asia	Guangdong, China	FSJG	2025	Hairy gourd	PV259286	2739	PV259310	2693
Asia	Guangdong, China	FSTG	2025	Melon	PV259285	2739	PV259309	2693
Asia	Guangdong, China	FSHL	2025	Bottle gourd	PV259284	2739	PV259308	2693
Asia	Guangdong, China	FSNG	2025	Pumpkin	PV259283	2739	PV259307	2693
Asia	Guangdong, China	FSSiG	2025	Luffa	PV259282	2738	PV259306	2692
Asia	Guangdong, China	STHL	2025	Bottle gourd	PV259281	2739	PV259305	2693
Asia	Guangdong, China	STFQ	2025	Tomato	PV259280	2739	PV259304	2693
Asia	Guangdong, China	STNG	2025	Pumpkin	PV259279	2739	PV259303	2693
Asia	Guangdong, China	STDG	2025	Wax gourd	PV259278	2739	PV259302	2692
Asia	Guangdong, China	STSG	2025	Luffa	PV259277	2739	PV259301	2693
Asia	Guangdong, China	STSiG	2025	Luffa	PV259276	2739	PV259300	2693
Asia	Guangdong, China	TSSiG	2025	Luffa	PV259275	2739	PV259299	2693
Asia	Guangdong, China	FTSiG	2025	Luffa	PV259274	2739	PV259298	2694
Asia	Guangdong, China	XXBG	2025	White melon	PV259273	2739	PV259297	2692
Asia	Guangdong, China	XXHG	2025	Cucumber	PV259272	2739	PV259296	2692
Asia	Guangdong, China	YJSiG	2025	Luffa	PV259271	2739	PV259295	2693
Asia	Guangdong, China	THSiG	2025	Luffa	PV259270	2739	PV259294	2678
Asia	Guangdong, China	ZCFQ	2025	Tomato	PV259269	2739	PV259293	2693
Asia	Guangdong, China	BYNG	2025	Pumpkin	PV259268	2739	PV259292	2693
Asia	Guangdong, China	BYSG	2025	Luffa	PV259267	2739	PV259291	2693
Asia	Guangdong, China	BYSiG	2025	Luffa	PV259266	2739	PV259290	2693
Asia	Shandong, China	SD	2025	Melon	PV083593	2739	PV083594	2692
Asia	Shandong, China	SD	2025	Cucumber	PV083591	2739	PV083592	2693

Note: ToLCNDV, Tomato leaf curl New Delhi virus; nt, nucleotides. Years are retained from the supplied dataset. GenBank accession numbers were verified against NCBI nucleotide records (https://www.ncbi.nlm.nih.gov/nuccore/).

Table S6. Primer sequences used for conventional PCR detection of ToLCNDV.

Primer	Sequence (5′–3′)
AC1-F	GAAGGAGGTTGAAAAATACGATCTA
AC1-R	CGGAGACGTTCTAGAATGGGGT
